# Postprandial C-Peptide to Glucose Ratio as a Marker of β Cell Function: Implication for the Management of Type 2 Diabetes

**DOI:** 10.3390/ijms17050744

**Published:** 2016-05-17

**Authors:** Yoshifumi Saisho

**Affiliations:** Department of Internal Medicine, Keio University School of Medicine, 1608582 Tokyo, Japan; ysaisho@keio.jp; Tel.: +81-3-5363-3797

**Keywords:** C-peptide, type 2 diabetes, β cell function, postprandial

## Abstract

C-peptide is secreted from pancreatic β cells at an equimolar ratio to insulin. Since, in contrast to insulin, C-peptide is not extracted by the liver and other organs, C-peptide reflects endogenous insulin secretion more accurately than insulin. C-peptide is therefore used as a marker of β cell function. C-peptide has been mainly used to assess the presence of an insulin-dependent state for the diagnosis of type 1 diabetes. However, recent studies have revealed that β cell dysfunction is also a core deficit of type 2 diabetes, and residual β cell function is a key factor in achieving optimal glycemic control in patients with type 2 diabetes. This review summarizes the role of C-peptide, especially the postprandial C-peptide to glucose ratio which likely better reflects maximum β cell secretory capacity compared with the fasting ratio in assessing β cell function, and discusses perspectives on its clinical utility for managing glycemic control in patients with type 2 diabetes.

## 1. Introduction

The incidence of diabetes is continuously increasing, and the current number of people with diabetes in Japan is estimated to be ~10 million. The number of people with diabetes in the world was estimated to be 415 million in 2015, and is expected to rise to 642 million in 2040 [1]. Five million people died from diabetes in 2015. Therefore, diabetes is an emerging problem in the world, and prevention and optimal treatment of diabetes are urgently needed. Most people with diabetes (>90%) are classified as having type 2 diabetes (T2DM). Although T2DM is characterized by obesity and insulin resistance, recent studies have revealed that a deficit of β cells is also a core problem in this type of diabetes [2]. Hence, the assessment of β cell function in clinical settings is important for the management of patients with T2DM as well as type 1 diabetes (T1DM).

This review summarizes the role of C-peptide, especially the postprandial C-peptide to glucose ratio, in assessing β cell function and discusses its clinical utility for managing glycemic control in patients with type 2 diabetes.

## 2. Diabetes and β Cell Function

Type 1 diabetes (T1DM) is characterized by β cell destruction mostly due to autoimmune attack [3]. β cell mass in patients with T1DM decreases, and, at the time of clinical diagnosis, functional β cell mass has usually already decreased to ~10% of normal [4]. Most patients with T1DM are in an insulin-dependent state and need multiple daily injections of insulin from the time of diagnosis.

On the other hand, T2DM is characterized by obesity and insulin resistance. Plasma insulin level is often higher than normal, so called hyperinsulinemia. Therefore, over the past several decades, T2DM has been considered a deficit of insulin action, in contrast to T1DM.

**Figure 1:**
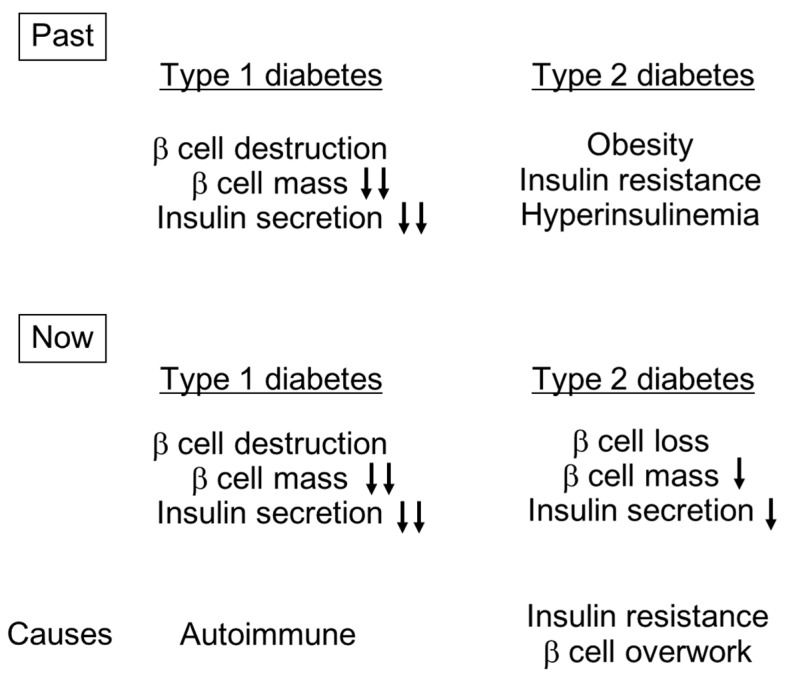
Concepts of the pathogenesis of type 1 and type 2 diabetes (T1DM and T2DM). Reduction in β cell mass and insulin secretion are greater in T1DM than T2DM.

However, since Butler *et al.* and other groups have reported reduced β cell mass in both lean and obese individuals with T2DM [5], it is now widely recognized that β cell deficit is a core feature of T2DM. The current concept of diabetes is shown in Figure 1. It is now appreciated that, despite the fact that the cause and extent of β cell loss are different between T1DM and T2DM, β cell deficit is a common pathogenetic feature of both types of diabetes. Therefore, assessment of β cell function is essential in both types of diabetes.

## 3. Measures of β Cell Function: C-Peptide

There are different measures of β cell function, as shown in Table 1. Acute insulin response (AIR) or AIRmax is the gold standard for assessment of β cell function [6]. However, as it requires a hyperglycemic clamp technique using an artificial pancreas, the use of these measures is limited to research purposes.

On the other hand, a marker obtained from a single blood sample would be more practical. C-peptide is a peptide connecting the A and B chains of insulin. In secretory granules of β cells, C-peptide is released from insulin by prohormone convertases. C-peptide is then secreted from β cells at an equimolar ratio to insulin, thereby reflecting endogenous insulin secretion. In contrast to insulin, C-peptide is not extracted by the liver and other organs, and the half life of C-peptide level in blood is longer than that of insulin (10–30 *vs.* 4 min). Therefore, C-peptide level reflects endogenous insulin secretion more accurately than insulin level.

In addition, the current insulin assay cannot distinguish endogenous insulin from exogenous insulin in patients who are receiving insulin therapy. Thus, C-peptide has the ability to measure endogenous insulin secretion in patients with diabetes irrespective of insulin therapy.

## 4. C-Peptide to Glucose Ratio

C-peptide can be measured in serum or urine. Serum and urinary C-peptide levels reflect the absolute amount of endogenous insulin secretion. However, since glucose itself is a major stimulus of β cells, insulin secretion is augmented by the higher glucose level seen in patients with diabetes. Thus, to assess β cell function, C-peptide level should be adjusted for glucose.

### 4.1. Clinical Utility of Postprandial C-Peptide to Glucose Ratio

Usually, C-peptide is measured after overnight fasting. As the plasma glucose level is relatively stable during fasting, insulin secretion is assumed to be stable. Thus, the assessment of β cell function using fasting samples is reproducible and more easily comparable within and between individuals. However, insulin secretion increases in a postprandial state. Since, in a postprandial state, not only higher plasma glucose level but also incretin effects stimulate insulin secretion, postprandial C-peptide level more likely reflects the maximal insulin secretory capacity compared with fasting C-peptide level, especially in patients with diabetes.

**Figure 2:**
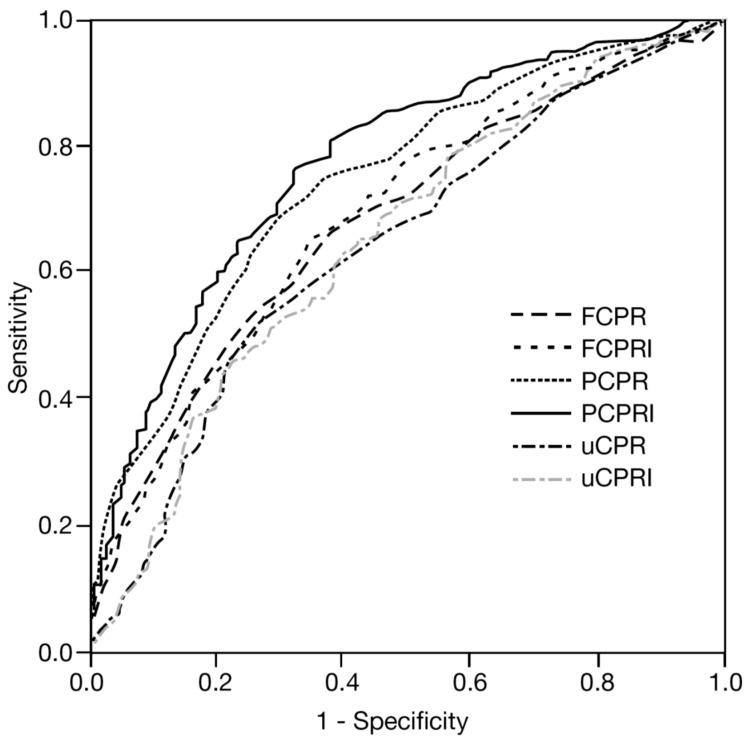
Comparison among C-peptide indices for predicting need for future insulin therapy (receiver operating characteristic (ROC) analysis). FCPR: fasting C-peptide; FCPRI: fasting C-peptide to glucose ratio; PCPR: postprandial C-peptide; PCPRI: postprandial C-peptide to glucose ratio; uCPR: 24 h urinary C-peptide; uCPRI: uCPR divided by fasting glucose level. Area under the curve (AUC) of PCPRI was the greatest (AUC: 0.779) among the indices, indicating the highest predictive value. Adapted from reference [8].

Most patients with T2DM eventually need insulin therapy for the control of hyperglycemia. We have examined the predictive utility of several C-peptide indices for the need for insulin therapy [8]. In 579 patients with T2DM admitted to our hospital, we measured fasting, postprandial and 24 h urinary C-peptide levels. Postprandial C-peptide was measured at 2 h after breakfast, and the need for insulin was defined as treatment with insulin therapy at the end of follow-up (mean 4.5 years). As a result, although all C-peptide indices were significantly associated with the need for insulin therapy, receiver operating characteristic (ROC) analysis revealed that postprandial C-peptide to glucose ratio (which we call “postprandial C-peptide immunoreactivity index; PCPRI”) was the best predictive marker for the need for insulin therapy among them (Figure 2) [8]. The same results were also obtained in a subanalysis of 190 patients with T2DM who were not treated with insulin at the time of admission [9]. The utility of PCPRI has also been confirmed in other cohorts. Fujiwara *et al*. have reported that PCPRI predicted the need for multiple daily insulin injections in patients with T2DM [10]. Lee *et al.* have reported that PCPRI was more useful for predicting treatment strategies such as oral antidiabetic agents and insulin therapy than other C-peptide indices [11].

Therefore, these findings suggest that among C-peptide indices, postprandial C-peptide rather than fasting C-peptide better reflects preserved β cell function. Recently, Okuno *et al*. have reported that postprandial, but not fasting, C-peptide to glucose ratio significantly correlated with disposition index calculated by glucose clamp technique, reflecting true β cell function adjusted for insulin sensitivity [12]. The authors speculated that PCPRI reflects postprandial glucose disposal in peripheral tissues. Meier *et al.* have also reported that PCPRI showed the strongest correlation with β cell mass compared with fasting C-peptide to glucose ratio and HOMA-β in patients who had undergone a pancreatectomy [13]. Thus, PCPRI likely predicts the maximal β cell secretory capacity and possibly more closely reflects β cell mass.

### 4.2. Cut-off Value of Postprandial C-Peptide to Glucose Ratio

In our cohort described above [8], the cut-off value of postprandial C-peptide (ng/mL) to glucose (mg/dL) ratio (×100) for prediction of the need for insulin therapy was 2.02, with 81% sensitivity and 63% specificity. On the other hand, PCPRI showing 80% specificity was 1.53 (61% sensitivity).

The relationship between patients with and without insulin treatment and PCPRI with cut-off values of 2.0 and 1.5, respectively are shown in Table 2. The positive predictive values (PPV) of insulin therapy were 79% and 84% with cut-off values of 2.0 and 1.5, respectively, indicating that ~80% of patients need insulin therapy using these cut-off values. On the other hand, the negative predictive values (NPV) of these cut-off values were 65% and 54%, respectively, indicating that even if PCPRI is above these cut-off values, 30%–40% of patients eventually need insulin therapy due to progressive loss of β cell function. Thus, PCPRI should not be used as the sole predictor but should be used as a supplemental marker to help physicians and patients understand the need for insulin therapy. Although PCPRI is an important factor to select the optimal anti-diabetic therapy, selection of medications should be decided individually, considering other factors such as age, duration of diabetes, HbA1c, body mass index (BMI), co-existing complications, risk of hypoglycemia, socio-economic status, and the patient’s preference.

### 4.3. Postprandial C-Peptide to Glucose Ratio and Glycemic Control

Patients with T2DM with lower PCPRI are more likely to be treated with insulin therapy. Nonetheless, in our cohort, these patients showed higher HbA1c and glycated albumin (GA) levels compared with those with higher PCPRI, suggesting poorer glycemic control in patients with T2DM and lower PCPRI despite the intensification of treatment [8]. Indeed, baseline PCPRI, rather than baseline HbA1c, was significantly, negatively correlated with HbA1c and GA after two years [14]. As these correlations were not changed after adjustment for medication, it is assumed that glycemic control in patients with low PCPRI is poorer than that in those with high PCPRI, irrespective of the treatment strategy. Although these studies were based on a retrospective cohort, an association between lower baseline β cell function and treatment failure has been also shown in prospective studies [15,16,17]. Recent studies have also shown that baseline C-peptide level is associated with the efficacy of incretin therapy [18,19]. We have shown that PCPRI is also negatively correlated with GA to HbA1c ratio, which reflects postprandial glycemic excursion and glycemic variability [20]; thus, it is suggested that patients with low PCPRI end up with poorer glycemic control with greater postprandial glycemic excursion and glycemic variability.

### 4.4. Measurement of C-Peptide Indices

As C-peptide and C-peptide to glucose ratio are useful for the diagnosis of T1DM, assessment of the presence of an insulin-dependent state and choice of medication, these measures need to be determined at the time of diagnosis. C-peptide and C-peptide to glucose ratio should also be measured thereafter when glycemic control worsens or when considering change in medication. In cases in which C-peptide and C-peptide to glucose ratio rapidly decline, the presence of T1DM or development of pancreatic cancer should also be considered and ruled out.

Although PCPRI appears to reflect the maximal β cell functional capacity more accurately compared with fasting C-peptide to glucose ratio, the timing of sampling (*i.e.*, 1 or 2 h after a meal) and the total calories and content of the meal may affect PCPRI. Thus, in clinical practice, fasting and postprandial C-peptide indices should be measured and assessed complementarily. Cut-off values of C-peptide indices for the need for insulin therapy with specificity of 80% are shown in Table 3 for comparison of these indices. However, these values were assessed in the Japanese population, and may not apply to other ethnicities, especially non-Asian populations.

The measurement of 24 h urinary C-peptide reflects total daily insulin secretion. However, since 24 h urine collection is not routinely conducted in daily clinical practice and the presence of urinary infection may cause underestimation of C-peptide level, serum C-peptide rather than urinary C-peptide is usually measured in the outpatient clinic. However, recently, the efficacy of 2 h postprandial urinary C-peptide to creatinine ratio obtained at home has been reported [21]. Thong *et al.* have reported that 2 h postprandial urinary C-peptide to creatinine ratio predicted the treatment response to liraglutide therapy [22].

Since C-peptide is excreted by the kidneys, serum C-peptide increases and urinary C-peptide decreases in patients with renal failure. Thus, the value of C-peptide in patients with renal failure should be carefully interpreted. In addition, C-peptide level may be overestimated in patients treated with insulin secretagogues such as sulfonylureas, glinides and incretin-related medication. C-peptide level may be underestimated due to glucose toxicity if marked hyperglycemia persists at the time of measurement. In this case, C-peptide level should be assessed again after correcting hyperglycemia.

## 5. Obesity Exaggerates Progressive Decline in C-Peptide Level in Patients with Type 2 Diabetes: β Cell Workload Hypothesis

T2DM is a progressive disease characterized by continuing loss of β cell function [23]. We have also observed that PCPRI is negatively correlated with the duration of T2DM [24]. In this cohort, when the patients were divided according to the presence or absence of obesity, we found that the decline in PCPRI with the duration of T2DM was exaggerated in obese subjects compared with lean subjects. It has also been reported that mean BMI of newly diagnosed T2DM patients has increased over the last decade [25]. However, the fasting insulin level in these patients continuously declined over the same period. These findings suggest that obesity itself accelerates the progressive decline in β cell function in patients with T2DM.

Insulin secretion is usually increased two- to three-fold to compensate insulin resistance in obese non-diabetic individuals [26]. On the other hand, only a 0% to 50% increase in β cell mass in obese non-diabetic individuals has been observed by histological analyses [27,28,29], implying that insulin secretion per β cell, *i.e.*, β cell workload, increases in the face of obesity.

**Figure 3:**
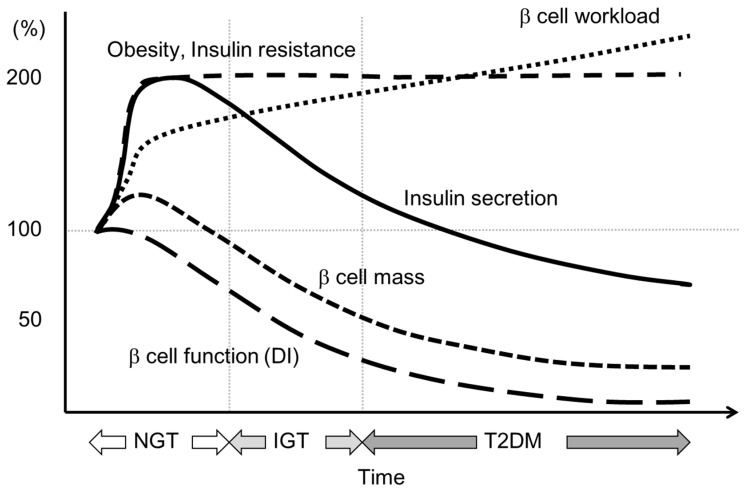
Hypothesis for change in β cell function and mass during development of abnormal glucose tolerance. The magnitude of the increased demand for insulin due to insulin resistance caused by excess caloric intake and physical inactivity exceeds the magnitude of β cell mass expansion, resulting in an increase in β cell workload. In individuals who are susceptible to T2DM, increased β cell workload may lead to β cell failure and the development of T2DM. Adopted from reference [2]. NGT: normal glucose tolerance; IGT: impaired glucose tolerance.

When the increase in β cell workload causes overwork of β cells, β cells may become dysfunctional and undergo apoptotic cell death through mechanisms such as oxidative stress, endoplasmic reticulum stress or amyloid deposition. Once a reduction in functional β cell mass occurs, the residual β cells then need to work harder to maintain normoglycemia. Eventually, when the reduction in functional β cell mass crosses the threshold, hyperglycemia develops. Hyperglycemia itself then causes a further increase in β cell workload and a further reduction in functional β cell mass through gluco(lipo)toxicity. This vicious cycle may explain the progressive nature of T2DM (Figure 3).

This hypothesis highlights the importance of early intervention and treatment of obesity to preserve and restore functional β cell mass in patients with T2DM.

## 6. Conclusions

This review summarizes the current knowledge of the clinical utility of C-peptide, especially postprandial C-peptide to glucose ratio, as a marker of β cell function in patients with T2DM. Since β cell deficit is a core defect in both T1DM and T2DM, C-peptide level should be assessed in patients with any type of diabetes. Assessment of β cell function by C-peptide and C-peptide to glucose ratio is useful not for only the diagnosis of T1DM but also for staging of T2DM and selection of the treatment strategy. C-peptide level can be measured in a fasting or postprandial state, and in serum or urine, and the combination of these indices will be useful. C-peptide indices are objective markers and will help physicians to discuss the need for treatment with patients. Such a decision-making process may improve patients’ adherence to therapy. Since postprandial C-peptide to glucose ratio (PCPRI) likely better reflects the maximal β cell functional capacity compared with the fasting value, and is easily measured in clinical settings, the assessment of PCPRI in conjunction with other clinical parameters may have the potential to improve patient care to preserve and recover residual β cell functional mass. To establish the role of measuring PCPRI and other C-peptide indices in the management of type 2 diabetes, further evaluation including study with a prospective design and cost-effective assessment is warranted.

## Figures and Tables

**Table 1:** Indices of insulin secretion and β cell function.

**Glucose Clamp-Based Indices** Gold standard but difficult to perform in clinical settings	
Acute insulin response (AIR)	Area under the curve (AUC) of plasma insulin during first 10 min of hyperglycemic clamp (200 mg/dL).
AIRmax	AIR with arginine stimulation. Reflects maximal insulin secretion.
Disposition index (DI)	Insulin secretion (AIR) adjusted for insulin sensitivity (M value). Reflects “true” β cell function.
**Glucagon Challenge Test**	
C-peptide or increase in C-peptide at 6 min	C-peptide ≤1.0 ng/mL or increase in C-peptide ≤0.5 ng/mL indicates insulin-dependent state [7].
**Indices Based on 75 g Oral Glucose Tolerance Test (OGTT) or Meal Tolerance Test (MTT)**	
Insulinogenic index (II)	Increment of insulin divided by increment of glucose during first 30 min of 75 g OGTT.
AUCinsulin/AUCglucose	AUC of insulin divided by AUC of glucose.
Oral DI	DI based on 75 g OGTT or MTT. Homeostasis model assessment of insulin resistance (HOMA-IR) or Matsuda index is used as insulin sensitivity index; *i.e.*, oral DI is calculated as II/HOMA-IR or AUCinsulin/AUCglucose×Matsuda index.
**Indices Based on Single Blood Sample** Simple and easy to use in clinical settings	
HOMA-β	360 × fasting insulin (mU/L)/(fasting glucose (mg/dL) − 63).
Fasting C-peptide	≤0.5 ng/mL indicates insulin-dependent state [7].
C-peptide to glucose ratio (CPRI)	C-peptide (ng/mL)/glucose (mg/dL) (×100). Assessed in fasting and postprandial states.
Proinsulin to insulin ratio (P/I ratio)	Reflects β cell health or exhaustion.
**Others**	
Urinary C-peptide	24 h urinary C-peptide reflects daily total insulin secretion. Recently, the utility of 2 h postprandial urinary C-peptide has been reported (See text).

**Table:** (**A**)

Parameter		Insulin Therapy		Total (*n*)
		+	‒	
PCPRI	<2.0	290	79	369
	≥2.0	74	136	210
Total (*n*)		364	215	579

Sensitivity 80%, Positive predictive value (PPV) 79%; Specificity 63%, Negative predictive value (NPV) 65%.

**Table:** (**B**)

Parameter		Insulin Therapy		Total (*n*)
		+	‒	
PCPRI	<1.5	213	40	253
	≥1.5	151	175	326
Total (*n*)		364	215	579

Sensitivity 59%, Positive predictive value (PPV) 84%; Specificity 81%, Negative predictive value (NPV) 54%.

**Table 3:** Cut-off values of C-peptide indices for need for insulin therapy with 80% specificity. The data are re-calculated from the original data of reference [8] (unpublished data).

Parameter	Cut-off Value	Specificity (%)	Sensitivity (%)
PCPRI	1.53	80	61
FCPRI	0.87	80	45
24 h urinary C-peptide (μg/day)	36.4	80	39

PCPRI; postprandial C-peptide to glucose ratio, FCPRI; fasting C-peptide to glucose ratio. Since PCPRI was measured at 2 h after breakfast during admission, these values may not apply to outpatient settings or other ethnicities. When C-peptide is expressed in nmol/L instead of ng/mL, PCPRI of 1.53 and FCPRI of 0.87 would be 0.51 and 0.29, respectively.

## Abbreviations

T1DM	type 1 diabetes
T2DM	type 2 diabetes
NGT	normal glucose tolerance
IGT	impaired glucose tolerance
FCPRI	fasting C-peptide to glucose ratio
PCPRI	postprandial C-peptide to glucose ratio
